# Artificial Placenta and Partial Ectogenesis for Extremely Preterm Infants: A Narrative Review

**DOI:** 10.7759/cureus.105357

**Published:** 2026-03-17

**Authors:** Tasneem A Abdulraheem

**Affiliations:** 1 Pediatrics, Jordanian Royal Medical Services, Amman, JOR

**Keywords:** artificial placenta, artificial womb, extreme prematurity, partial ectogenesis, periviable birth

## Abstract

Extremely preterm births at the edge of viability are associated with high mortality and significant long-term morbidity. This is largely due to the sudden and involuntary shift from fetal placental support to neonatal lung breathing, accompanied by systemic organ injury. Between 22 and 24 weeks of gestation, the fetal lungs, brain, and metabolic regulatory systems are not biologically prepared for extrauterine life. As a result, extremely preterm infants are highly vulnerable to ventilator-induced lung injury, oxygen toxicity, hemodynamic instability, and inflammatory cascades that contribute to bronchopulmonary dysplasia and neurological injury. Modern neonatal intensive care can provide respiratory, hemodynamic, nutritional, and supportive care; however, it cannot fully reproduce fetal physiology. Artificial placenta and artificial womb technologies, often described as partial ectogenesis, aim to provide extracorporeal gas exchange through the umbilical vessels while maintaining fluid-filled lungs in a closed, thermoregulated, amniotic-like environment to support fetal physiology after delivery. Preclinical ovine models using pumpless arteriovenous circuits and sealed fluid environments have demonstrated the feasibility of supporting fetuses at developmental stages comparable to human periviability for prolonged periods while maintaining stable fetal circulation. However, major translational barriers remain, including reliable umbilical vascular access, development of ultra-low-resistance oxygenators that function at fetal flows and pressures, hemocompatibility and anticoagulation management, infection control during prolonged extracorporeal support, and the inability to replicate the placenta’s endocrine and metabolic functions necessary for normal fetal growth and organ maturation. The ethical implications are also substantial. Partial ectogenesis may alter how clinicians and society approach viability, influence thresholds for resuscitation and periviability counseling, complicate consent in high-stress emergency settings, and generate distributive justice concerns if access remains limited to highly specialized centers. This narrative review summarizes the physiological rationale, major preclinical evidence, translational challenges, and ethical considerations surrounding artificial placenta and partial ectogenesis technologies for extremely preterm infants.

## Introduction and background

Periviable birth (22-25 weeks’ gestational age) continues to pose significant ethical and clinical challenges in neonatology. Although survival is possible, it is often associated with a substantial risk of severe morbidity and long-term neurodevelopmental impairment (NDI) [1,2]. Population-level studies have shown that survival without NDI has gradually improved over time. Nevertheless, the absolute rates remain low and vary considerably between centers, indicating that outcomes depend not only on technological advances but also on institutional practices and clinical decision-making [3].

Current obstetric and neonatal guidelines emphasize individualized counseling that considers gestational age, fetal condition, maternal circumstances, and family values when navigating situations characterized by substantial uncertainty [4]. In this context, innovations that reduce injury, not only mortality, are of considerable clinical and ethical importance.

One of the major challenges in extremely preterm infants is the premature transition from placental gas exchange to pulmonary respiration. Initiation of breathing and exposure of structurally immature lungs to oxygen, together with the distending pressures required for mechanical ventilation, can activate inflammatory pathways and disrupt normal alveolar and vascular development. This process contributes to bronchopulmonary dysplasia (BPD) and other long-term respiratory complications [5,6].

The developing brain is also highly vulnerable during this period. Encephalopathy of prematurity results from a combination of impaired and abnormal brain maturation associated with hypoxia-ischemia, inflammation, hemodynamic instability, and infection [7]. Consequently, even carefully optimized neonatal intensive care may expose extremely premature infants to physiological stresses that would normally be avoided in the intrauterine environment.

The concepts of the artificial placenta and the artificial womb aim to reconsider neonatal care by preserving fetal physiology outside the uterus immediately after birth. Rather than forcing an immediate transition to air breathing, these systems attempt to extend “gestation-like” extrauterine support for a limited period before a planned transition to conventional neonatal care [8]. This approach is often described as partial ectogenesis, distinguishing it from the theoretical concept of complete ectogenesis beginning at conception [9].

## Review

Concepts and definitions

Artificial placenta systems attempt to maintain fetal physiology outside the uterus using extracorporeal gas exchange and a controlled fluid environment [10]. The concept of partial ectogenesis refers to maintaining gestation outside the uterus for a limited period rather than the entire duration of pregnancy [11]. Ethical discussions have emphasized that this approach represents a transitional form of gestation rather than complete ectogenesis [12].

BPD is now recognized as a disorder of disrupted lung development influenced by inflammation, oxygen toxicity, and mechanical ventilation, particularly in extremely premature infants [2]. Technologies designed to preserve fetal physiology and avoid early pulmonary ventilation may therefore help reduce the lung injury associated with extreme prematurity.

The term artificial womb is used in the literature to describe a broader extrauterine support platform that combines extracorporeal gas exchange with a regulated fluid-filled environment resembling the amniotic sac, including temperature control and fluid management [10,12].

Partial ectogenesis refers to gestation occurring outside the uterus for a period shorter than the full duration of pregnancy [7]. In the near-term clinical context, it describes temporary extrauterine support for extremely preterm fetuses or neonates rather than complete gestation outside the body [7,12].

The ethical importance of these concepts extends beyond terminology. Romanis (2018) argued that artificial womb technology should not be understood simply as an extension of neonatal intensive care, because the supported entity may occupy an intermediate conceptual state that is not fully captured by the terms “fetus” or “newborn” [7]. Similarly, De Bie et al. (2023) discussed the ethical relevance of this transitional state in relation to future clinical deployment of artificial womb technology [12].

Preclinical evidence

What Can Be Learned from Large Animal Models?

The most influential proof-of-concept study was reported by Partridge et al. (2017), who demonstrated that an extrauterine support system could sustain extremely premature lambs equivalent to approximately 23-24 weeks of human gestation for up to four weeks [3]. The system consisted of a pumpless arteriovenous circuit driven by the fetal heart, a low-resistance oxygenator, and a closed sterile fluid environment designed to preserve fetal physiology. Importantly, the lambs maintained stable hemodynamics and patterns of fetal circulation, suggesting that prolonged extrauterine support may be feasible if resistance, oxygenation, and environmental conditions are carefully controlled [3].

Subsequent studies have expanded this preclinical foundation. Usuda et al. (2023) reported support of extremely preterm ovine fetuses at the border of viability for up to 336 hours while maintaining systemic circulation, although reduced somatic and organ growth were observed [11]. These findings are highly significant because they suggest that successful gas exchange and hemodynamic stability alone are insufficient; preservation of fetal growth and developmental maturation remains an essential translational requirement [11].

Related work has also examined organ-specific development during extrauterine support. For example, Baumgarten et al. (2020) reported that the EXTra-uterine Environment for Neonatal Development supported normal intestinal maturation and development, suggesting that at least some organ systems may continue to mature under suitable extrauterine conditions [8].

Together, these animal studies demonstrate that prolonged support of fetal physiology outside the uterus is possible in principle. At the same time, they also show that physiologic success must be judged not only by short-term survival but also by preservation of growth, organ maturation, and longer-term developmental integrity [3,8,11].

Physiological explanation of ectogenesis in decreasing morbidity

Reduction of Lung Injury

The predominant early injury pathway in extreme prematurity involves the lung. BPD is now recognized as a disorder of disrupted lung development influenced by inflammation, oxygen toxicity, and mechanical ventilation, especially in the most immature infants [2]. By bypassing pulmonary gas exchange and maintaining fluid-filled lungs during a highly vulnerable developmental period, artificial placenta systems aim to reduce ventilator-induced lung injury and oxygen-mediated disruption of alveolar and vascular development [3,10]. In theory, preservation of fetal circulation and lower pulmonary blood flow may also reduce hemodynamic instability and systemic inflammatory signaling.

Potential Neuroprotection

A second potential benefit is neuroprotection. The preterm brain is highly susceptible to injury from inflammation, infection, hypoxia-ischemia, and circulatory instability [6]. White matter injury may not be immediately apparent clinically, yet it remains a major contributor to later NDI in survivors of extreme prematurity [6]. By maintaining fetal circulation and avoiding mechanical ventilation during this vulnerable period, artificial placenta systems may theoretically reduce oxidative stress, inflammatory activation, and fluctuations in cerebral perfusion. However, these possible neuroprotective effects remain hypothetical and require confirmation in further preclinical and eventual clinical studies [10-12].

Translational challenges

Umbilical Vascular Access

Umbilical-based access remains a major limitation. After delivery, the umbilical cord is typically clamped, and cannulation of the umbilical vessels may cause endothelial injury, vasospasm, hemorrhage, or thrombosis [3,10]. Unlike conventional extracorporeal membrane oxygenation (ECMO), where peripheral vascular access may be obtained after postnatal stabilization, fetal-mode extrauterine support generally requires immediate catheter placement in the umbilical vessels before pulmonary ventilation is initiated. This makes the perinatal workflow a tightly coordinated obstetric-neonatal procedure rather than a sequential handoff [10,13].

Circuit Resistance and Oxygenator Design

Circuit resistance is a primary engineering concern in pumpless arteriovenous systems. Because flow depends on fetal cardiac output and circuit resistance, even small increases in resistance may significantly impair perfusion and oxygen delivery. The oxygenator must therefore support effective gas exchange at fetal pressures and flow rates while maintaining extremely low resistance. Cannulas, tubing, and the priming volume of the circuit must also be specifically optimized for fetal physiology [3,10]. These requirements strongly suggest that successful clinical systems will require purpose-built devices rather than scaled-down neonatal ECMO circuits [10,13].

Hemocompatibility and Anticoagulation

Hemocompatibility and anticoagulation represent another major challenge. As with all extracorporeal systems, blood-surface interactions can trigger coagulation and inflammatory cascades, creating the competing risks of thrombosis and hemorrhage [9,10]. This issue is especially critical in extremely preterm patients, who are already at high risk of bleeding, including intracranial hemorrhage. Key translational priorities include reducing device-induced activation through improved biomaterials and coatings, designing clot-resistant oxygenators, developing appropriate monitoring strategies for very small blood-volume systems, and identifying anticoagulation strategies that maintain circuit patency without causing major bleeding [10,13].

Infection Control

Infection prevention is also a major concern during prolonged extrauterine support. Although chronic ovine support studies have not reported the same degree of device-related infection risk expected in clinical settings, human use would involve greater manipulation, a more complex microbiological environment, and highly vulnerable patients [10,13]. Therefore, strict aseptic technique, sterile fluid systems, close surveillance for infection, and robust system design are essential. Infection control should be considered a core design challenge rather than an afterthought.

Endocrine and Metabolic Insufficiency

One of the most difficult obstacles is replicating the non-respiratory functions of the placenta. The placenta is not only a gas exchange organ, but it is also a critical metabolic and endocrine interface that regulates nutrient transfer, growth signaling, and hormone production. Long-duration ovine studies showing reduced growth and lower concentrations of markers such as insulin-like growth factor-1 and albumin indicate that developmental physiology may remain suboptimal even when oxygenation and perfusion are maintained [11]. This limitation is central to clinical translation because the ethical justification for artificial placenta technology depends not merely on prolonging survival, but on improving long-term outcomes through preservation of normal development [10-12].

Transition to Postnatal Life

Finally, transitioning off the system represents a planned “second birth.” Partial ectogenesis does not eliminate the transition to postnatal life; it postpones it. Clinical translation will require clear protocols regarding when and how to transition from fluid-filled lungs to air breathing, manage ductal physiology, stabilize thermoregulation and autonomic function, and introduce feeding [10,13]. This transition phase may itself carry substantial risk of hemodynamic instability and organ injury and therefore requires systematic preclinical study.

Ethical considerations

From a regulatory and ethical perspective, this field requires careful governance before first-in-human application. De Bie et al. (2023) emphasized that ethical debate has often focused on futuristic ideas of complete ectogenesis rather than near-term neonatal applications of artificial womb technology [12]. They argued that early clinical use should be approached as medical research, not routine therapy, to minimize therapeutic misconception and protect informed consent [12].

The ethical implications are closely tied to the concept of viability. If artificial placenta or artificial womb technologies substantially improve outcomes at 22-23 weeks of gestation, clinicians and societies may need to reconsider current thresholds for intervention. However, viability is not determined by technology alone; it is also shaped by local resources, social values, support systems, disability perspectives, and parental preferences [5,13].

Consent is particularly difficult in periviability because decisions are made under intense time pressure and emotional stress. Romanis (2018) cautioned against presenting artificial womb technology as simply a “better incubator,” as doing so may overstate the certainty of benefit and obscure the experimental nature of the intervention [7]. Consent discussions must therefore clearly distinguish such technology from standard care and explicitly address uncertainty, risks, and investigational status [7,12].

Justice is another major concern. These systems are likely to be expensive, technically complex, and initially available only in highly specialized centers. Limited access could widen existing disparities in neonatal outcomes unless equity is considered early in policy development and implementation [12,14,15].

Finally, conceptual framing matters. Romanis (2018) highlighted the importance of terminology in shaping ethical and legal discourse [7], while De Bie et al. (2023) similarly emphasized the ethical significance of how this supported entity is understood [12]. These conceptual debates are not merely semantic; they may affect regulation, parental expectations, consent processes, and broader public discussion.

Strengths and weaknesses of the evidence base

Most of the published evidence so far comes from preclinical studies in sheep. These models offer significant insights into cardiovascular dynamics and circuit engineering; however, they inadequately address the complexities of human neurodevelopment, immune vulnerability, and the practical challenges encountered during emergent delivery and neonatal resuscitation. Standardization and comparison of studies are deficient as various research groups work with different platforms and methodologies. Importantly, long-term functional status remains ill-defined, and failure to thrive in extended life-support paradigms underscores that programming endocrine and metabolic disruption is presumably incomplete for human translation. In the era of modern neonatal care, when the primary ethical emphasis is on surviving with a good quality of life, both artificial womb and artificial placenta technologies will need to show more than just a physiological possibility but also a clear potential for reducing morbidity to be viable options as clinical therapies.

## Conclusions

The use of artificial placenta and partial ectogenesis are new methods that could have a big impact on the health of very premature babies by improving fetal physiology and stopping unwanted transitional physiology from happening in babies who are getting ready for airway gas exchange modes of gas exchange. Clinical translation is still limited because of several technical and clinical problems, even though experimental models have a lot of promise. More research and a closer look at the ethics will be needed to determine if these technologies can safely improve survival and lower long-term illness in this group of people.
